# Supplementary material for: Long Term Assessment of Anti-SARS-CoV-2 Immunogenicity after mRNA Vaccine in Persons Living with HIV
Source: Vaccines (Basel). 2023 Nov 22;11(12):1739. doi: 10.3390/vaccines11121739 (PMC10747871; doi:10.3390/vaccines11121739)
Supplement: Supplementary file 1 [file vaccines-11-01739-s001.zip › vaccines-2674177-supplementary.pdf]

Supplementary Material

Supplementary Table S1. Number of marker values used at each of the time points from the 314 participants

	Vaccination schedule times			
**Response**	**T1[a]**	**T2[b]**	**T3[c]**	**T4[d]**
**nAbs WD614G**				
Overall	**172**	**314**	**195**	**135**
LCD4	35	56	40	27
ICD4	59	120	80	60
HCD4	78	138	75	48
**nAbs Omicron**				
**BA.1**	**79**	**103**	**103**	**80**
Overall	25	33	34	25
LCD4	30	37	36	31
ICD4	24	33	33	24
HCD4				
**IFN-gamma**				
Overall	**130**	**299**	**188**	**108**
LCD4	27	53	41	24
ICD4	39	116	76	42
HCD4	64	130	71	42

a) One month after II dose
b) Pre III dose
c) Two weeks after III dose
d) Six months after III dose or before IV dose

Supplementary Figure S1.

Adjusted absolute mean Omicron BA.1 nAbs values over T1-T4 from fitting a mixed linear model (left panel) and box-plots (right panel) of the raw data by CD4 count groups. Y-axis reports nAbs values expressed in Log_2 (cut off 5.32 log_2);X-axis T1-T4 timepoints . The timepoints are: T1: 2 months after PVC , T2: 4 months after PVC , T3: 2 weeks after 3rd dose , T4: 5 months after 3rd dose.

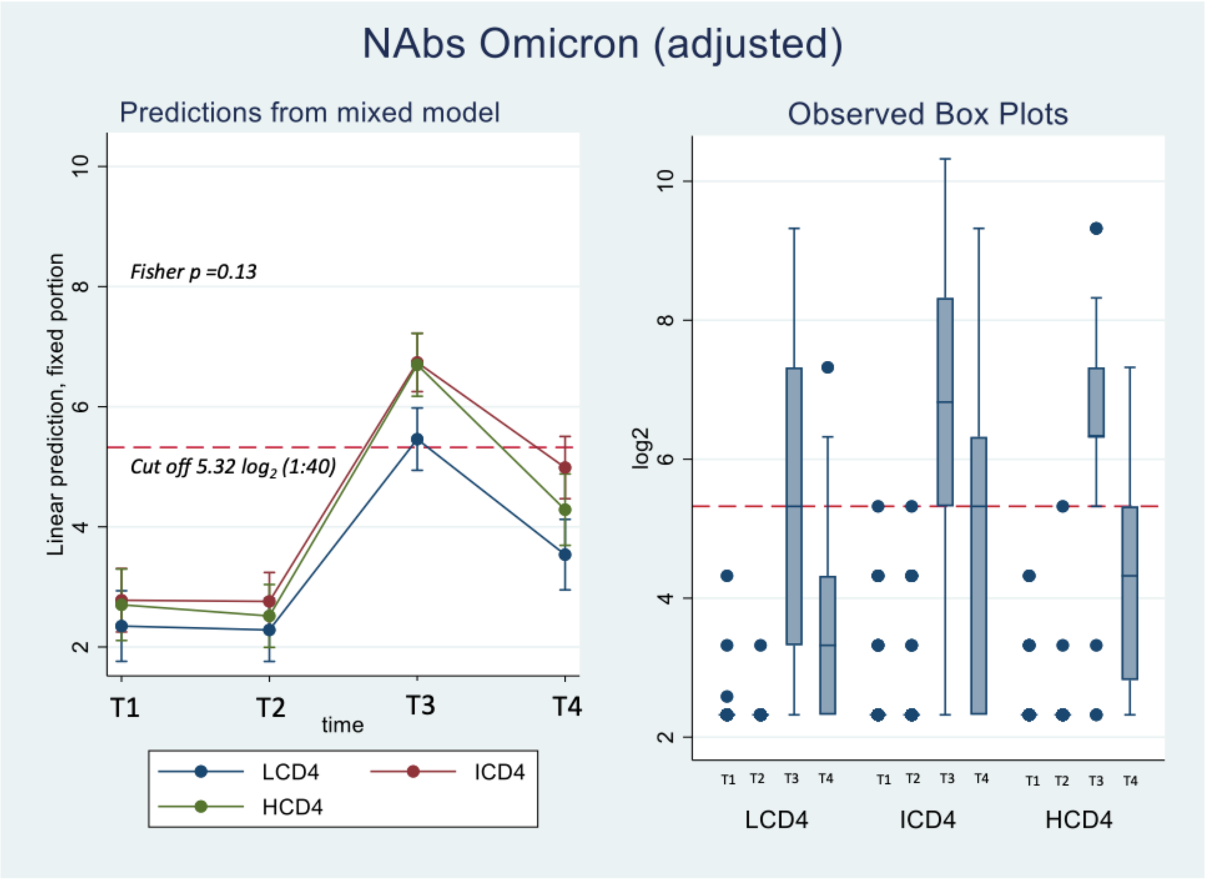

Supplementary Table S2A. Adjusted absolute mean Omicron BA.1 nAbs values over T1-T4 from fitting a mixed linear model (left panel) and mean differences (right panel) by CD4 count groups.

	Adjusted means				Adjusted difference in means				
**Response**									
	**T1 95% CI**	**T2 95% CI**	**T3 95% CI**	**T4 95% CI**	**T1 95% CI**	**T2 95% CI**	**T3 95% CI p-value**	**T4 95% CI p-value**	**p-value***
**NAbs Omicron**									
CD4 group									0.125
HCD4	2.7 (2.1, 3.3)	2.5 (2.0, 3.0)	6.7 (6.2, 7.2)	4.3 (3.7, 4.8)	0	0	0	0	
ICD4	2.8 (2.2, 3.3)	2.7 (2.3, 3.2)	6.8 (6.3, 7.2)	5.0 (4.5, 5.5)	0.0 (-0.7, 0.8)	0.2 (-0.5, 0.9)	0.1 (-0.7, 0.8)	0.7 (-0.1, 1.5)	
					0.918	0.540	0.888	0.076	
LCD4	2.4 (1.8, 2.9)	2.3 (1.8, 2.8)	5.5 (4.9, 6.0)	3.5 (2.9, 4.1)	-0.4 (-1.2, 0.5)	-0.2 (-1.0, 0.5)	-1.2 (-2.0, -0.5)	-0.7 (-1.6, 0.1)	
					0.399	0.551	0.001	0.093	

*F-test type 3 interaction p-value

&Adjusted for gender age and CD4 count nadir

Supplementary Table S2B. Adjusted mean changes from T2 in Omicron BA.1 nAbs values over T1-T4 from fitting a mixed linear model (left panel) and mean differences (right panel) by CD4 count groups.

	Adjusted& mean changes from T2$				Adjusted& difference in mean changes from T2$			
**Response**								
	**T1 95% CI**	**T2 95% CI**	**T3 95% CI**	**T4 95% CI**	**T1 95% CI p-value**	**T3 95% CI p-value**	**T4 95% CI p-value**	**p-value***
**NAbs Omicron**								

CD4 group								0.162
HCD4	0.4 (-0.2, 0.9)	REF	4.3 (3.8, 4.8)	1.9 (1.4, 2.5)	0	0	0	
ICD4	0.1 (-0.4, 0.6)	REF	4.0 (3.5, 4.5)	2.1 (1.6, 2.6)	-0.3 (-1.1, 0.4)	-0.3 (-1.0, 0.4)	0.2 (-0.5, 1.0)	
					0.395	0.401	0.576	
LCD4	-0.1 (-0.6, 0.5)	REF	3.1 (2.6, 3.6)	1.2 (0.6, 1.7)	-0.4 (-1.3, 0.4)	-1.2 (-1.9, -0.5)	-0.8 (-1.6, 0.0)	
					0.276	<.001	0.063	
$One month after 3rd vaccine dose								
*F-test type 3 interaction p-value								
&Adjusted for gender age and CD4 count nadir								

Supplementary Table S3. Matrix of Spearman rho correlation coefficients

	nAbs at T1	nAbs at T3	IFN-γ at T1
nAbs at T1			
nAbs at T3	0.72<br>P<0.0001		
IFN-γ at T1	0.48<br>P<0.0001	0.37<br>P<0.0001	
IFN-γ at T3	0.24<br>P=0.002	0.29<br>P<0.0001	0.68<br>P<0.0001

Supplementary Figure S2 A. Correlation at time T1

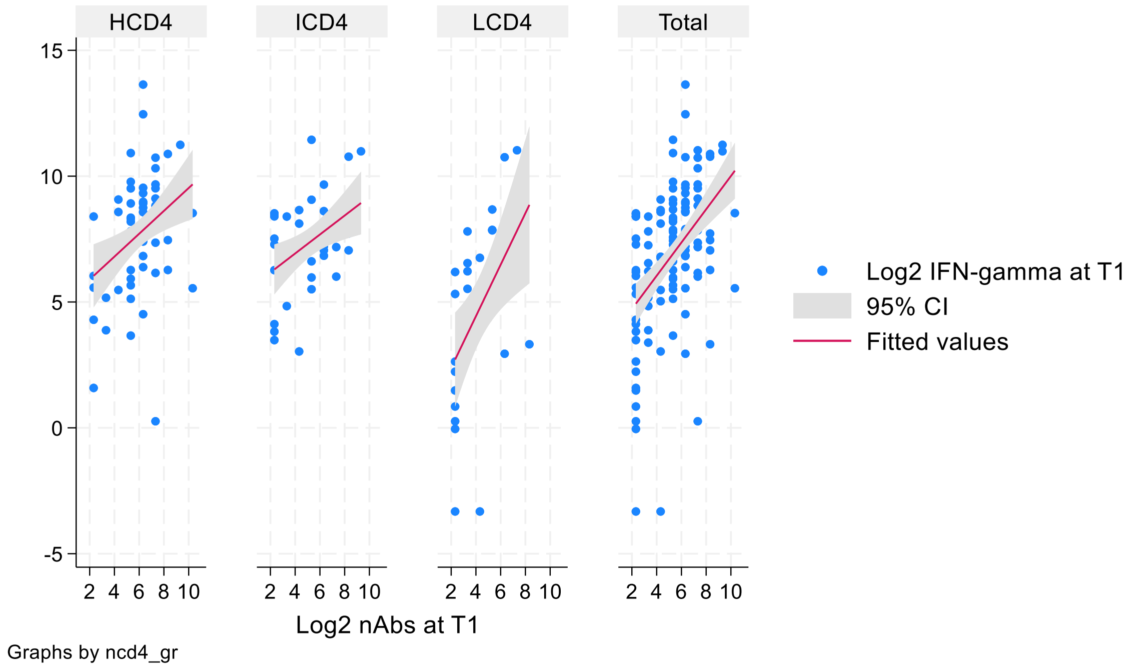

Supplementary Figure S2B. Correlation at time T3

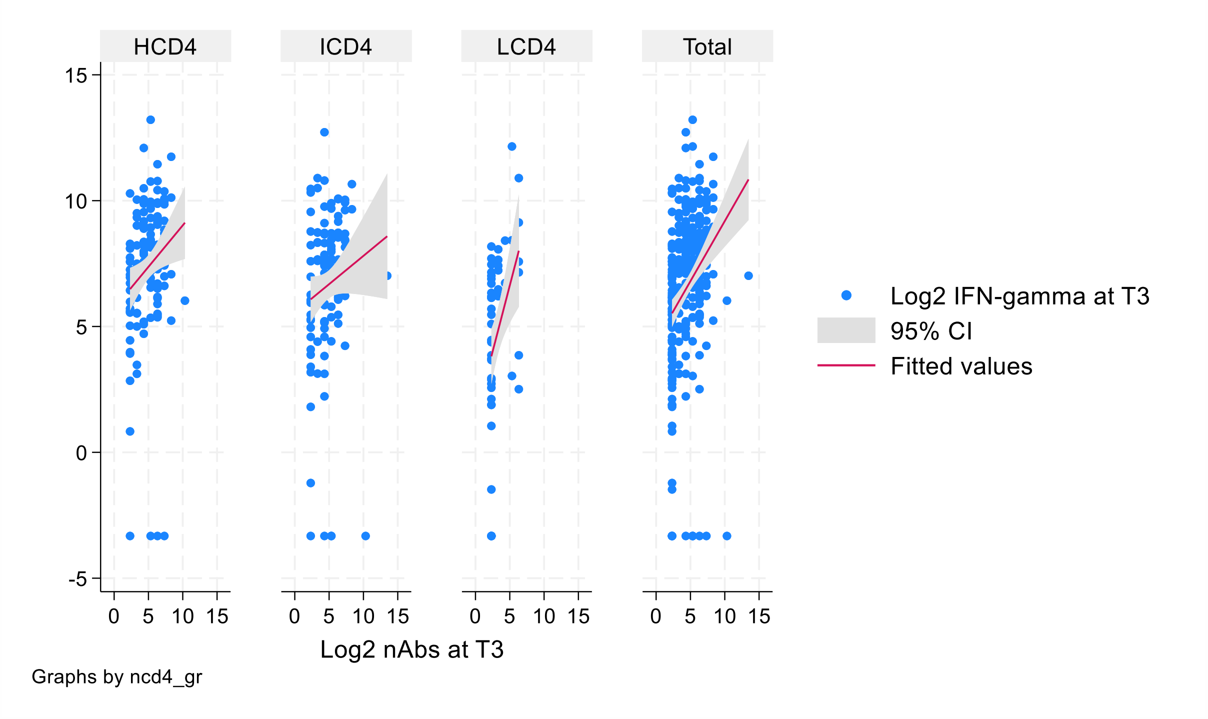

HCD4
ICD4
LCD4
Total
15
10
5
0
-5
0 5 10 15
Log2 nAbs at T3
Log2 IFN-gamma at T3
95% CI
Fitted values
Graphs by ncd4_gr
